# Face validity of the EQ health and wellbeing instrument (EQ-HWB) in Hong Kong

**DOI:** 10.1186/s41687-026-01075-4

**Published:** 2026-05-08

**Authors:** Eliza Lai-Yi Wong, Annie Wai-Ling Cheung, Amy Yuen-Kwan Wong, Zhihao Yang

**Affiliations:** 1 JC School of Public Health and Primary Care, Faculty of Medicine, The Chinese University of Hong Kong, Hong Kong SAR, China; 2 Centre for Health Systems and Policy Research, JC School of Public Health and Primary Care, Faculty of Medicine, The Chinese University of Hong Kong, Hong Kong SAR, China; 3 Health Services Management Department, Guizhou Medical University, Guiyang, China

**Keywords:** EQ-HWB, Face validity, Cultural adaptation, Quality of life measurements, Economic evaluations

## Abstract

**Background:**

The EuroQol Health and Wellbeing (EQ-HWB™) measures quality-of-life (QoL) across healthcare and social care settings. Its framework integrates physical, social, and psychological domains, shaped by cultural influences. Despite global validation, evidence supporting its applicability in Asian contexts, particularly in Hong Kong, is limited. This study evaluated the face validity of the Traditional Chinese version of the EQ-HWB among Hong Kong adults, focusing on its conceptual adequacy and comprehensiveness in the local setting.

**Methods:**

A qualitative study was conducted between August 2023 and August 2024, 30 participants were recruited through quota sampling stratified by age, sex, and education levels. The sample included healthy individuals, those with chronic conditions, and informal caregivers. Cognitive debriefing interviews using the ‘think-aloud’ method assessed the comprehensibility, relevance, and appropriateness of the 25-item EQ-HWB. Thematic analysis focused on seven predetermined questions addressing clarity, conceptual meaning, and social desirability.

**Results:**

Most EQ-HWB items were well-understood, though linguistic ambiguities (e.g., idioms like “exhausted”) and overlapping concepts (e.g., “no support from others” vs. “accepted by others”) posed challenges. Participants reported difficulties distinguishing response scales (e.g., “occasionally” vs. “sometimes”) and suggested simplifying examples and refining scales. No social desirability bias was observed, with confidentiality highlighted as essential for honest responses.

**Conclusion:**

The EQ-HWB exhibits strong face validity in Hong Kong but requires cultural and linguistic adjustments to improve clarity. Recommendations include revising ambiguous phrasing, clarifying conceptually similar items, and optimizing response options. These refinements will enhance the tool’s suitability for local economic evaluations and cross-sectoral wellbeing assessments.

**Supplementary Information:**

The online version contains supplementary material available at 10.1186/s41687-026-01075-4.

## Highlights

Authors should identify 2–3 “Highlights” that illustrate the paper’s contribution to the field. These bulleted statements should address:

i. What is already known about the topic?

The EQ-HWB has been globally validated as a comprehensive measure of Quality of Life (QoL) across healthcare and social care settings. While previous research has established its psychometric properties and conceptual framework in Western populations, evidence regarding its applicability in Asian contexts remains limited. In particular, the Traditional Chinese version for use in Hong Kong had not been evaluated. Prior studies focused on item selection rather than evaluating the final instrument’s performance in local settings.

ii. What does the paper add to existing knowledge?

This study makes significant contributions as the first to rigorously assess the face validity of the EQ-HWB Traditional Chinese version Hong Kong. It identifies key cultural-linguistic challenges in the local adaptation, including ambiguous terminology and overlapping concepts. These findings provide valuable insights and evidence-based recommendations for refinements to enhance instrument’s clarity, cultural relevance and measurement precision in Chinese populations.

iii. What insights does the paper provide for informing healthcare-related decision making?

The study offers valuable insights for healthcare policymakers by demonstrating how cultural adaptation can improve a measurement tool’s validity. Our findings support the EQ-HWB’s potential for cross-sectoral wellbeing assessments in Hong Kong while highlighting necessary modifications. These adaptations will enable more accurate QoL measurements in economic evaluations, ultimately leading to better-informed resource allocation decisions in Chinese populations across healthcare, public health and social care settings.

**Supplementary Information:**

The online version contains supplementary material available at 10.1186/s41687-026-01075-4.

## Introduction

Following the publication of the E-QALY (Extending the QALY) project papers, there has been growing global interest in utilising and validating the EuroQol Health and Wellbeing (EQ-HWB) instrument across diverse jurisdictions, including Hong Kong [1, 2]. The EQ-HWB was developed to address limitations of existing health utility measures, such as the EQ-5D and SF-36, which primarily focus on health status, including physical and mental health. Other generic HRQoL measures, such as WHOQOL-BREF, additionally include social and environmental domains. However, these measures may overlook important aspects of health and wellbeing relevant to social care users, carers and certain patient populations. The EQ-HWB goes beyond health and incorporates domains that reflect overall life satisfaction and capability, aiming to capture a more holistic view of well-being. This distinction is important because it positions the EQ-HWB as complementary to, rather than interchangeable with, existing generic HRQoL tools. While HRQoL tools are rooted in health-related constructs and are often used for health outcomes assessment and cost-utility analysis, the EQ-HWB is designed for broader policy evaluation, including interventions that impact well-being beyond health care. This approach aims to enable more consistent comparisons of benefits across healthcare, social care and public health sectors [3].

The EQ-HWB is a novel instrument which comprise of 25-item across seven domains – feelings and emotions, cognition, self-identity, coping, physical sensation, relationships, and activities. The abbreviated EQ-HWB-S includes 9 items representing a subset of the full version and is available for economic evaluations [4]. As the tool is still in the experimental phrase, accessible for use is not available to public which limits external opportunities for validation. To date, the EQ-HWB has undergone rigorous translation from English (UK version) into several languages, including simplified Chinese, Spanish and German [1]. The translation process was followed according to the standardised EuroQol translation protocol to ensure both linguistic and cultural appropriateness across languages. This involved forward and backward translation, complemented by cognitive debriefing procedures to verify clarity and relevance [1].

However, evidence on the applicability of the EQ-HWB in Asian contexts remains limited. Previous E-QALY face validity studies conducted in mainland China primarily focused on item selection rather than evaluating the final instrument. These studies were not optimal for several reasons, approximately half of the items were modified after the development stage, and only the final items were assessed from an initial pool of 96 items grouped into seven domains. The performance of the finalised EQ-HWB instrument was not comprehensively evaluated. Therefore, further research on its content and face validity is warranted for Chinese-speaking populations, including Hong Kong.

Hong Kong presents a unique sociolinguistic environment characterised by diglossia, in which spoken Cantonese is the dominant vernacular while written communication primarily uses standard written Chinese. This linguistic duality can influence how respondents interpret and comprehend survey instruments translated from English into Traditional Chinese. While written Chinese is widely understood, its formal register often differs significantly from colloquial Cantonese expressions used in daily life. Consequently, certain idiomatic phrases or abstract concepts in the translated EQ-HWB may not align with respondents’ spoken language norms, potentially affecting clarity and perceived relevance. Recognising this diglossic context is essential for evaluating face validity, as comprehension challenges may arise not from conceptual inadequacy but from sociolinguistic mismatches between written forms and everyday speech.

The present study addresses this gap by examining the face validity as part of the overarching project aimed at assessing its content validity of the EQ-HWB using the experimental version among laypeople in Hong Kong. Content validity refers to the extent to which the items on a questionnaire accurately reflect the scope of the research question and comprehensively cover the construct of interest. In other words, it assesses whether the indicators represented by the scale’s items adequately reflect the underlying dimensions it tends to measure [5]. For multi-attribute utility instrument (MAUI) such as the EQ-5D and EQ-HWB-9, content validity pertains to the appropriateness of the classification system in representing health status. Face validity, refers to the apparent relevance and appropriateness of an instrument, ensuring that it appears to measure what it is intended to measure [6]. This aspect is crucial for enhancing acceptability and response rates in future applications. This study aims to evaluate the face validity that focuses on the clarity, comprehensibility, and cultural appropriateness of the EQ-HWB among the adult population in Hong Kong, with particular consideration given to informal caregivers.

## Methods

### Study design

A qualitative study design was adopted and carried out between August 2023 and August 2024. This study focuses on the face validity of the Traditional Chinese Hong Kong version of the EQ-HWB to ensure that it measures what it is intended to measure by evaluating its comprehensiveness, appropriateness and items relevancy of the EQ-HWB in the target Hong Kong population. By examining the face validity of the two EQ-HWB instruments (short and long version) (Supplementary Table 1), it helps to ensure that the instrument appears to measure what it is intended to measure, which should help with future acceptability and response rates. The experimental version of the EQ-HWB (Traditional Chinese version for Hong Kong) used in this study has been modified after this study completed, the current version is specified as ‘modified version’ [7].

### Sampling and data collection

Quota sampling was employed to recruit respondents aged 18 years or above from the general population of Hong Kong, ensuring diversity in age, gender, education, health conditions, and caregiving experience. Inclusion criteria required participants to be native Cantonese speakers residing in Hong Kong. Participants who were unable to speak Cantonese, read written Chinese, or provided informed consent were excluded from the study. Although education within the local school system was not mandatory, all participants were familiar with the local linguistic context. Cognitive Debriefing, as an essential component of the translation process, involves testing translated materials with a representative sample of the target population to ensure clarity, cultural appropriateness, and accurate comprehension. The study sample comprised three groups: individuals with physical or mental illnesses, healthy individuals, and informal caregivers, with 10 respondents in each group.

Semi-structured qualitative interviews were conducted in four steps: (1) introduction and warm-up questions; (2) exploring the concepts of ‘health’ and ‘well-being’; (3) to complete the EQ-HWB questionnaire (Traditional Chinese Hong Kong version) in cognitive debriefing interview using the ‘think-aloud’ method; and (4) using the QQ-10 questionnaire to assess participants’ views on the use of EQ-HWB [8]. The interview process consisted of four steps, while this study focused specifically on Step 3 for assessing face validity. In Step 3, upon participants’ completion of the EQ-HWB questionnaire, discussion on each item of the EQ-HWB was followed based on the predetermined set of seven questions to evaluate participants’ understanding and interpretation of the instrument (Supplementary Table 2) [5].

### Study procedure

This study explored the face validity of EQ-HWB in Hong Kong and was conducted in parallel with the Mainland China research team. For this study, the EQ-HWB was adapted into Traditional Chinese from the English version, which included full translation and content validation translation process according to the standardised EuroQol translation protocol [1]. Study protocol and topic guide were aligned with those used in the Italian study [5]. Interviewer training including sharing experiences and interview techniques was provided by the Mainland China team. Minor adjustments of the adopted topic guide were employed due to cultural and language differences for adaptation. Before the commencement of actual data collection, five pilot interviews were conducted to improve interviewing techniques and data quality. Based on the pilot findings and collaboration discussions, the Hong Kong team adjusted the interview ordering within step 2 of the topic guide to reduce potential confusion and response bias. Despite these refinements, both teams adhered to the same quota sampling criteria and core interview steps.

Participants were instructed to use the think-aloud method to complete the EQ-HWB items. Prior to the main task, a familiarisation session was involved where participants were asked to describe their activities in the last 7 days, articulating every thought that comes to mind. Once they are familiar with the think-aloud method, they will proceed to complete the EQ-HWB, verbalising their thoughts for each item. The interview may use prompts to encourage participants to express their thoughts as needed, including the seven predetermined questions as shown in Table 2.

### Qualitative analysis

The recorded data from the 30 interviews was transcribed verbatim and examined to assess face validity (Step 3). Participants feedback on the EQ-HWB items were entered individually for qualitative analysis and thematic coding to maintain accuracy, ensuring data quality and completeness. For face validity, respondents’ feedback from cognitive debriefing interviews was collected using the think-aloud method. Seven pre-determined questions guided the evaluation of item clarity, conceptual meaning, appropriateness of labels and examples, and potential for social desirability bias. Participants’ feedback were categorised using the E-QALY face validity framework and organised according to these seven questions to support structured analysis and interpretation. Interview transcripts were thematically coded and analysed in NVivo (version 15.0) [9], and feedback was summarised in a matrix mapping identified problems across items and domains. These codes were used to group similar feedback across different questions and items. This inductive approach combines content analysis with thematic synthesis to ensure both question specific and cross-item patterns were captured. The analysis focused on identifying ambiguities, uncertainties, and difficulties in item interpretation, as well as suggestions for improvement.

## Results

### Sample characteristics

In total, 30 respondents participated and completed the face-to-face individual interviews. Respondents age range between 18 and 69 years, and 43.3% (13/30) had chronic conditions. Gender distribution was equally split and 36.7% (11/30) had tertiary or above education attainment. Sample characteristics are presented in Table 1.

**Table 1:** Demographic characteristics

	*N*	(%)
Sex		
Male	15	(50.0)
Age		
18–29	6	(20.0)
30–39	5	(16.7)
40–49	6	(20.0)
50–59	6	(20.0)
60–69	7	(23.3)
Health Conditions		
Healthy	17	(56.7)
Chronic condition	13	(43.3)
Informal caregiver	15	(50.0)
Religion		
No religion	20	(66.7)
Christian	8	(26.7)
Buddhism	2	(6.7)
Education		
*Tertiary or above	11	(36.7)

*Tertiary education refers to Bachelor degree or above

### EQ-HWB face validity results

The results of EQ-HWB face validity in Hong Kong are reported according to the sequence of the predetermined questions used in the cognitive debriefing interview (Table 2).

**Table 2:** A set of predetermined questions used in the cognitive debriefing interview

	Predetermined questions
1	Whether the item is easy to understand and answer to?
2	What does the item mean?
3	Whether the items labels and descriptors are relevant and appropriate?
4	Whether the examples given are appropriate?
5	Whether it makes respondent embarrassing (happy to respond it honestly)?
6	Whether it could result social desirable response?
7	Whether the labels and descriptors are important to health and wellbeing?

Face validity analysis revealed that three out of 25 items received no feedback including item 5 ‘self-care’, item 10 ‘trouble remembering’ and item 20 ‘feel good about yourself’. Respondents reported difficulties were found by choosing between the severity and frequency response scales. The items that generated the most feedback were item 18 (‘unable to cope with day-to-day life’), item 19 (‘accepted by others’), item 17 (‘had no control over your day-to-day life’) and item 13 (‘feel unsafe’), with 20, 18, 17, and 16 respondents reported with raising concerns, respectively. These feedback primarily focused on the clarity of item wording and the appropriateness of the labels and examples provided. Additional minor feedback was reported for items 6, 8, 9, 16, 24, 25 (‘problems with sleep’, ‘lonely’, ‘no support from others’, ‘hand nothing to look forward’, ‘pain’ and ‘discomfort’), each noted by at least three respondents. Respondents repeatedly reported difficulty in distinguishing between item 9 and item 19. Identified problems related to participant responses are discussed and categorised in the following sections, with each measure addressed individually including but not limit to ambiguities, relevance, embarrassing items, complicated or confusing items and resulting in social desirability responses. Matrix of response with identified problems across the seven predetermined questions is presented in Table 3.

**Table 3:** Matrix of response identified problems by participants across the 25-item EQ-HWB using the 7 predetermined questions framework

Item	1	2	3	4	5	6	7	8	9	10	11	12	13	14	15	16	17	18	19	20	21		22	23		24		25	
Domain	AC					PS		RE		CO		FE					AU		RE	SI			PS						
Participant																													
P1									1																				
P2																	1											1	
P3																		1											
P4				1		1		1	1				1			1	1	1	1							1		1	
P5									1				1				1	1	1		1								
P6													1					1	1									1	
P7						1					1							1											
P8																	1	1	1										
P9	1												1				1	1	1										
P10								1								1	1	1	1									1	
P11	1	1						1					1				1	1	1										
P12															1		1	1	1				1	1		1		1	
P13						1							1	1															
P14									1				1					1											
P15																		1											
P16													1																
P17																		1	1							1			
P18							1																			1			
P19						1																							
P20																	1	1	1	1									
P21						1			1				1				1	1	1					1		1		1	
P22													1			1	1	1	1		1							1	
P23		1	1						1			1	1	1			1		1							1		1	
P24				1					1				1				1		1							1		1	
P25						1							1				1	1	1							1			
P26													1				1	1	1									1	
P27							1						1				1	1	1							1		1	
P28												1		1				1								1			
P29									1								1		1									1	
P30													1																
Total	2	2	1	2	0	6	1	3	8	0	1	2	16	3	1	3	17	20	18	0		2	1		2		10		12

AC: Activity; PS: Physical Sensation; RE: Relationship; CO: Cognition; FE: Feeling and Emotion; AU: Autonomy; SI: Self-identity

### Questions 1 – Whether the item is easy to understand and answer to?

The majority of respondents reported that overall was easy to understand and items labels were straightforward; while some indicated that it was moderately easy but most of the items are clearly understandable (7 respondents). In addition to the 7 respondents, few respondents reported that the EQ-HWB was not an easy task and considered challenging to comprehend or respond to. The underlying reasons for these challenges faced by respondents were categorised into three aspects: (1) ambiguities, (2) uncertainties, and (3) difficulties (5 respondents). Individual items that were difficult to understand were examined under Question 2 ‘Understanding the meaning of items’. The identified themes were organised according to each of the seven predetermined questions.

#### Ambiguity

Some respondents reported that the items were ambiguous, despite still being understandable in terms of the Chinese characters themselves or familiarity with the words presented. Some of the items were found duplicate where respondent reported *(“it’s understandable*,* but sometimes some items seem a bit vague or ambiguous” P11)*,* (“some of the questions are relatively easy to understand*,* but other tend to be more repetitive” P22).*

However, some questioned whether they had interpreted the meanings correctly but responded based on their initial feelings, such as with the items ‘exhausted’, ‘lonely’, ‘unsafe’, ‘frustrated’, ‘sad or depressed’ and ‘had no control’. Confusion also arose when items contained too many examples, especially when considering each situation individually *(“Some items are not easy to understand and quite vague. For example*,* day-to-day activities – working and shopping are totally different. I believe they are all different things and they are distinct to each other” P23).*

#### Uncertainty

Several items were identified as complicated and uncertain, primarily involving item 1–4. Similar ambiguity was observed, this aspect appears to be related to the provided examples, leading the respondents with uncertainty. Participants struggled to assess the levels of difficulty, particularly those who wear glasses, hearing aid or walking stick; however, difficulties were also reported by individuals who do not use such aids *(“I don’t need hearing aid*,* but if it’s too noisy at times*,* I found it difficult to hear or might not be clear. If several people were talking together*,* I found it a bit difficult” – uncertain whether they are considered as experiencing any difficulties P11)*,

#### Difficulty

Some respondents found it difficult to answer due to the process of thinking in terms of the items’ wording itself, while others found that *(“It’s not easy*,* sometimes you really have to think”*,* “There are many questions require you to think and you won’t be able to respond immediately”*,* “You need to consider the extent or the level of what you’re seeing*,* especially since the recall period of the last 7 days makes it difficult to answer” P6)*.

### Question 2 – What does the item mean?

#### Struggling in understanding

Most of the respondents found the EQ-HWB items understandable and were able to convey their meaning, while others struggled to identify the words or did not recognise the words themselves. Item 1–3, 4, 7, 8, 11, 12, 14–15, 17 had received the most feedback from the respondents.

For Item 1–3, respondents stated that *(“I thought that I am required to imagine or visualise as I need to use walking stick even if I do not need it usually” – caused confusion P23).*

Item 4 ‘day-to-day activities’ received numerous feedback from respondents indicating that the provided examples were misleading. For instance, some respondents did not consider working as part of ‘day-to-day activities’, and they perceived the question *(“was asking about the difficulties I have encountered at work or with specific task” P23*,* P24*,* P25)*.

Respondents indicated that some items, such as idioms in Chinese, for example, item 7 ‘feeling exhausted 筋疲力歇’ is not widely recognised and is deemed unclear. They questioned *(“whether it refers to having no strength or what is it about? Noting that some people might be very clear*,* but perhaps others are not very sure about it” P27)*. Similar to item 11 ‘have trouble concentrating or thinking clearly’. It was raised out that the two items are distinct to each other which makes it difficult to response. Respondents reported *(“Maybe for myself*,* I might wonder whether it was asking about if I could concentrate during work. Or if I could not focus due to sickness which affects my daily work” – unable to answer for having two distinct items in a question P28).*

#### Conceptual clarity issues

Several items were found to be unclear in their literal meaning and difficulty was encountered in understanding the conceptual meaning of these items (Item 13, 16, 17, 18, 21). For instance, item 16 ‘had nothing to look forward to’ was found ambiguous *(“whether there is thing you look forward to” P14 or “whether I have things but have no expectations about it” P16).* Similarly, item 18 *‘day-to-day life *日常生活’ is (“very general term” P23).

Item 13 and item 17 were reported as problematics by numerous respondents, as their meanings were unclear and there were difficulties in comprehending the underlying concepts. 16 out of 30 respondents have feedback on item 13 and reported that *(“thinking that it gives a sense of security – mentally P24”*,* “Because of this questionnaire ‘Health and Wellbeing’*,* if no examples were given*,* I would think about my own condition and those kinds of things internally rather than physical harm by others” P30)*. Item 17 ‘no control over your day-to-day life’ also have various meaning to different persons *(“I think control refers to the decision of whether there is thing to do*,* or how you want to do it*,* depends on many factors that determine whether you are able to complete it or not” – it is not something one can decide to respond to this question P26)*.

#### Semantic similarity

Item 12, 14 and 15 were generally perceived as similar or were considered difficult for respondents to distinguish clearly: *(“it seems almost the same”*,* “Anxiety*,* I can be understood as feeling frustrated”*,* “But I think frustrated is similar to feeling sad. I found it difficult to understand as items 14 and 15 are fairly similar. it also seems similar to feeling depressed” P13*,* P23*,* P28).*

Item 19 ‘accepted by others’ received feedback from 18 respondents that it had similar meanings to item 9 ‘no support from others’ but only asked in an opposite way (P30). Some might consider ‘being accepted’ refers to *(“sense of belongings*,* feeling that you are being yourself” P22).* Similar difficulties were observed that *(“item 16 ‘had nothing to look forward’ and item 21 ‘do the things wanted to’ are quite similar. Essentially*,* they are the same*,* just asked in opposite ways” P23).* However, respondents raised out that they were uncertain about item 21 was referring to complete a task at present or goal of achievement (P24).

### Question 3 – Whether the items labels and descriptors are relevant and appropriate?

#### Appropriateness of labels

Regarding the appropriateness of the item labels and response options, respondents expressed concerns that, particular among older adults *(“distinguishing between the terms ‘occasionally’ and ‘sometimes’ may be challenging*,* potentially leading to misunderstandings” P24).* Some participants reported *(“difficulty comprehending the phrase ‘slight*少許*difficulty’ and it was actually quite similar to ‘some *有些*difficulty” P11*,* P24*,* P27).* Item 23 and 25 assess the severity of ‘pain’ and ‘discomfort’, respectively, and were identified as potentially redundant, given their conceptual similarity and repetitive. The primary distinction between ‘discomfort’ and ‘pain’ lies in severity, with ‘pain’ regarded as a more severe condition compared to ‘discomfort’ (P14, P22, P24, 27, 28). It is also noted that the frequency response options inherently convey severity levels.

Overall, respondents indicated that the response options for several items are ambiguous, particularly for item 6 ‘sleep’, item 7 ‘exhausted’ and item 8 ‘lonely’. They suggested that employing a numerical scale or specifying the number of days, rather than categorical options such as ‘none’ to ‘most of the time’, would be more appropriate (P20, P24, P26, P28).

### Question 4 – Whether the examples given are appropriate?

The majority of respondents reported that the provided examples were appropriate and contributed to a clearer understanding of the items. However, some participants found the examples overly detailed, which potentially hindered comprehension and created confusion. Nine respondents (P4, 6, 9, 10, 22, 23, 25, 26 and 28) reported inaccuracies in the item descriptions within the provided examples, with some indicating that these examples helped facilitate their understanding of the item.

#### Misleading examples

Examples for Item 4, ‘day-to-day activities’ and 17 ‘no control over day-to-day life’, received the most feedback concerning inaccurate descriptions, leading to ambiguity among the respondents. It was noted that ‘working’ is distinct from the other examples and should not be classified as an ‘activity’.

#### Mixed impact of examples on item comprehension

In contrast to the inaccuracies reported in Sect.  4.1, item 13 ‘unsafe’ received more positive feedback suggesting that the provided examples helped facilitate respondents’ understanding. However, it is also noted that the item ‘unsafe’ was unfamiliar to most respondents, with uncertainty regarding whether it referred to emotional or physical safety (16 participants reported difficulties to the literal meaning). While some respondents felt that the examples for item 17 had little relevance to the item label, with two stating that the examples can be deleted *(“because I think everyone has many different feelings about themselves*,* adding descriptions may even cause confusion” P22)*. Others reported that *(“when it comes to choosing options for doing things*,* it is not necessarily related to this item*,* which do not consider as having no control over your day-to-day life” P23).*

### Question 5 – Whether it makes respondent embarrassing?

Almost all respondents reported experiencing no embarrassment for any items while completing the questionnaire, allowing them to respond honestly and comfortably.

### Question 6 – Whether it could lead to socially desirable responses?

#### Confidentially and emotional sensitivity

Although no items were identified as exhibiting social desirability bias, respondents emphasised the importance of confidentiality and self-reporting, particularly for emotionally sensitive items. Potential concerns were noted for items related to emotions, such as item 12 ‘anxious’, 15 ‘feeling sad or depressed’, and item 8 ‘lonely’.

### Question 7 – Whether the labels and descriptors are important to health and wellbeing?

#### Comprehensive coverage with contextual gaps

Respondents generally agreed that the EQ-HWB instrument is appropriate and sufficiently comprehensive to capture key aspects of health and wellbeing. However, suggestions were made to include additional domains such as work satisfaction, physical activity and healthy diets, financial difficulties, and social relationships, as these were considered important components for evaluating health and wellbeing in the local context.

## Discussion

This study represents the first evaluation of the face validity of the EQ-HWB in the local Chinese context in Hong Kong. Our findings demonstrate that the EQ-HWB shows strong validity as a measure of health and wellbeing, reflecting its overall relevance and appropriateness. Most items were perceived as clear and meaningful; however, some challenges emerged, particularly regarding linguistic nuances and conceptual interpretation. Identified problems were mainly linked to ambiguous wording and difficulties in understanding certain underlying concepts, indicating the need for cultural and linguistic refinements.

These findings align with previous research underscoring the significance of face validity in instrument development [1, 4, 5, 10, 11]. Nevertheless, participant feedback revealed challenges in item comprehension, primarily related to ambiguous wording, such as the use of Chinese idioms expressions, and difficulties in interpreting the intended conceptual meaning.

The face validity analysis of the EQ-HWB instrument underscores both strengths and areas for refinement. The high level of clarity observed for most items suggests that the questionnaire is generally comprehensible, aligning with prior research emphasizing the importance of clear wording in Health-related Quality of Life measures [1]. However, the feedback indicates that certain items may benefit from revision to enhance clarity and reduce ambiguity, particularly those that elicited the most concerns. The underlying reasons for these challenges faced by respondents were categorised into three aspects: ambiguities, uncertainties, and difficulties in understanding the items likely due to individual perception differences, inappropriate examples or linguistic complexity in the local context.

The three items that received no feedback – ‘self-care’, ‘trouble in remembering’ and ‘feel good about yourself’ appear to be well-understood, likely due to their straightforward language and universal relevance. Conversely, the items received multiple feedback highlight specific area where item wording could be refined. For instance, the phrases used for items related to “coping” or “control” might be interpreted variably depending on individual resilience or cultural context, which could influence responses. Clarifying the scope of what coping or control entails or providing more specific examples might mitigate this ambiguity [5].

The recurrent confusion between item 9 ‘no support from others’ and item 19 ‘accepted by others’ illustrates the subtle distinction between social support and social acceptance. Although the meaning of the two items are conceptually related, these constructs are not identical. Providing clearer differentiation within the items or including explicit definitions could enhance respondent understanding and improve response validity. Challenges related to uncertainty and difficulty further highlight the cognitive demands placed on respondents, which may stem from linguistic complexities and differences between literal and conceptual meaning in the local context. Items incorporating multiple examples or broad scenarios, such as those addressing day-to-day activities or social interactions, may benefit from simplification or the removal of unnecessary examples [12]. Breaking down composite items into more specific, targeted questions could also reduce respondent burden and improve response accuracy.

The greatest challenges reported by respondents concerned the response scales, particularly the distinction between the frequency and severity levels, which warrant further attention. Participants found it difficult to differentiate between these response options, potentially introducing measurement errors or response bias. This challenge appears to stem largely from linguistic complexities in the Chinese context, where scale descriptors are often vague and used interchangeably. Future iterations should consider providing clearer guideline on response scaling, such as specifying the number of days for frequency, to promote more consistent and accurate responses.

The current findings align with existing literature emphasising the importance of cultural and linguistic adaptation in health measurement tools [1, 6, 12–14]. Ambiguities and uncertainties often stem from linguistic nuances or cultural differences in interpreting health-related concepts. Although this study employed cognitive interviewing techniques during instrument development adapted from protocols used in other jurisdiction which help to identify and address potential issues, ensuring that items are both linguistically appropriate and culturally relevant, some challenges persisted. Furthermore, findings from the assessment of social desirability bias, including measures of embarrassment, indicated minimal concern regarding respondents providing socially acceptable answers. This is likely due to the assurance of privacy and confidentiality, which underscores the preference for self-reporting methods. Maintaining strict confidentiality protocols and emphasising respondent anonymity are essential strategies. Such measures can help enhance the validity of responses by fostering a more comfortable environment for honest reporting. Despite the consistent perceptions of QoL, the study identified areas requiring attention to enhance the understandability and comprehensibility of the EQ-HWB. Recommendations include improving translation accuracy, refining examples, simplifying items, and ensuring appropriate response options.

## Conclusion

This study provides the first evidence of face validity for the EQ-HWB in Hong Kong, confirming its overall appropriateness while identifying areas for improvement. The EQ-HWB demonstrates promising clarity and relevance, and targeted revisions based on respondent feedback can further enhance its validity and reliability. Although most items were well understood, challenges related to linguistic ambiguity, conceptual overlap, and response scale interpretation highlight the importance of cultural and linguistic adaptation. Addressing these issues, such as refining idiomatic expressions, reducing cognitive burden by simplifying examples, and improving clarity in frequency and severity scales will enhance measurement precision and respondent acceptability. Incorporate iterative testing and cognitive interviewing in future validation efforts will be essential to ensure the instrument accurately captures the multidimensional nature of health and wellbeing in Chinese-speaking populations, thereby serving as a robust tool for economic evaluations. Further refinement and cultural adaptation remain necessary to maximise its effectiveness in this setting. Future research should also explore psychometric properties and test the instrument in broader healthcare and social care contexts to establish its reliability and comparability internationally. 

## Supplementary Information

Below is the link to the electronic supplementary material.

Supplementary Material 1

## Data Availability

The datasets used and/or analysed during the current study are available from the corresponding author on reasonable request.
