# Supplementary material for: Face validity of the EQ health and wellbeing instrument (EQ-HWB) in Hong Kong
Source: J Patient Rep Outcomes. 2026 May 8;10:108. doi: 10.1186/s41687-026-01075-4 (PMC13319299; doi:10.1186/s41687-026-01075-4)
Supplement: Supplementary file 1 — Supplementary Material 1 [file 41687_2026_1075_MOESM1_ESM.docx]

**Suppl. Table 1. Items used in the Experimental Version of the EQ-HWB**

| Domain name | Item name | EQ-HWB Experimental version |
| --- | --- | --- |
|  |  | In the last 7 days: |
| Activity | Vision | Q1. How much difficulty did you have seeing (using, e.g. glasses or contact lenses if you normally use them)? |
| Activity | Hearing | Q2. How much difficulty did you have hearing (using, e.g. hearing aids if you normally use them)? |
| Activity | Mobility | *Q3. How much difficulty did you have getting around inside and outside (using e.g. a walking stick or if you normally use them)? |
| Activity | Activities | *Q4. How much difficulty did you have doing day-to-day activities (e.g. working, shopping, housework)? |
| Activity | Self-care | Q5. How much difficulty did you have washing, using the toilet, getting dressed, eating, or caring for your appearance? |
| Physical Sensation | Sleep | Q6. Did you have problems with your sleep? |
| Physical Sensation | Exhausted | *Q7. Did you feel exhausted? |
| Relationship | Loneliness | *Q8. Did you feel lonely? |
| Relationship | Support | Q9. Did you feel that people did not support you? |
| Cognition | Memory | *Q10. Did you have trouble remembering? |
| Cognition | Cognition | Q11. Did you have trouble concentrating or thinking clearly? |
| Feeling and Emotion | Anxiety | *Q12. Did you feel anxious? |
| Feeling and Emotion | Safety | Q13. Did you feel unsafe? (e.g. fear of falling, physical harm, abuse) |
| Feeling and Emotion | Frustration | Q14. Did you feel frustrated? |
| Feeling and Emotion | Sad | *Q15. Did you feel sad/depressed? |
| Feeling and Emotion | Hopeless | Q16. Did you feel you had nothing to look forward to? |
| Autonomy | Control | *Q17. Did you feel you had no control over your day-to-day life? (e.g. had no choice to do things or have things done for you as you like and when you wanted) |
| Autonomy | Coping | Q18. Did you feel unable to cope with day-to-day life? |
| Relationship | Relationship | Q19. Did you feel accepted by others? (e.g. felt like you were able to be yourself and that you belonged) |
| Self-identity | Self-identity | Q20. Did you feel good about yourself? |
| Self-identity | Self-identity | Q21 Could you do the things you wanted to do? |
| Physical Sensation | Pain frequency | Q22. Did you have physical pain? |
| Physical Sensation | Pain severity | *Q23. How much physical pain you had in the last 7 days? |
| Physical Sensation | Discomfort frequency | Q24. Did you have physical discomfort? (e.g. feeling sick, breathless, itching (not including pain) |
| Physical Sensation | Discomfort severity | Q25. How much physical discomfort you had in the last 7 days? |

*Indicated as EQ-HWB-S short version.

**Suppl. Table 2. EQ-HWB qualitative interview topic guide**

| Step 1 | 1. Introduction |
| --- | --- |
| Step 2 | - 1. Warm up and background questions |
|  | - 1. Conceptual exploration of ‘Wellbeing’, ‘Health’ and the relationship between them |
| Step 3 | - 1. Think-aloud method familiarisation |
|  | - 1. Complete the EQ-HWB using think-aloud method |
|  | - 1. Evaluation on EQ-HWB items based on the predetermined questions |
| Step 4 | 1. QQ-10 questionnaire |
